# Temporal Variation and Human Host Predominance in *Aedes aegypti* from Coastal and Western Kenya: Insights from Pooled Blood Meal Metagenomics

**DOI:** 10.3390/pathogens14050505

**Published:** 2025-05-21

**Authors:** Kavinya Mwendwa, Francis Mutuku, Sammy Wambua, Makenzi Nzaro, Bryson A. Ndenga, Kennedy Agoi, Angelle D. LaBeaud, Carren Bosire

**Affiliations:** 1 Department of Pure and Applied Sciences, Technical University of Mombasa, Mombasa 80100, Kenya; mwendwakavinya@gmail.com (K.M.); makenzinzaro@gmail.com (M.N.); agoikennedy91@gmail.com (K.A.); 2 Department of Environment and Health Sciences, Technical University of Mombasa, Mombasa 80100, Kenya; fmutuku73@gmail.com; 3 Department of Biological Sciences, Pwani University, Kilifi 80108, Kenya; sammywambua@gmail.com; 4 Pwani University Biosciences Research Centre (PUBReC), Pwani University, Kilifi 80108, Kenya; 5 Research and Conservation Support Society [RECOURSE], Kilifi 80108, Kenya; 6 Kenya Medical Research Institute, Centre for Global Health Research, Kisumu 40100, Kenya; bndenga@yahoo.com; 7 Department of Pediatrics, Division of Infectious Diseases, Stanford University School of Medicine, Stanford, CA 94305-5139, USA; dlabeaud@stanford.edu

**Keywords:** *Aedes aegypti*, blood meal source, arboviral diseases, group test

## Abstract

*Aedes aegypti* is the primary vector of arboviral diseases such as dengue, chikungunya, yellow fever, and Zika, posing significant global health and economic challenges. The effective control of this mosquito species requires understanding its seasonality, feeding behavior, and ecological dynamics. Modern molecular techniques, such as amplicon metagenomic sequencing, provide insights into vector–host interactions and feeding patterns. This study investigated the temporal variation of *Ae. aegypti* abundance and its blood meal sources in coastal and western Kenya over 16 months. A total of 64,360 mosquitoes were collected, with *Ae. aegypti* comprising 10.9% (7035/64,360). Coastal sites had a higher proportion (64.7%) of *Ae. aegypti* than western Kenya. Seasonal variation in abundance was observed, with peaks during the long rainy season and decline during the dry season. Blood meal analysis identified 15 vertebrate hosts, with humans being the primary source (86.6–95.9%). Other hosts included domestic animals such as turkey, sheep, cow, goat, and chicken. These findings highlight the role of rainfall in arboviral disease transmission and *Ae. aegypti*’s strong preference for human hosts. Additionally, this study demonstrates the cost-effectiveness of group testing for identifying blood meal sources, with implications for public health interventions.

## 1. Introduction

*Aedes aegypti* is the principal vector of dengue virus and several other medically important arboviruses, including chikungunya, yellow fever, and Zika virus. Its remarkable adaptability to diverse environments—ranging from urban to semi-urban and rural settings—makes it the most efficient vector for dengue transmission [1,2]. Within these environments, *Ae. aegypti* utilizes a variety of blood and sugar sources to meet its reproductive and metabolic demands [1].

Over the past two decades, dengue fever has expanded significantly and now poses a growing global public health threat [3], including in Africa [4], where its burden is expected to increase under projected climate change scenarios [5]. In Kenya, dengue is now considered endemic, with more than seven outbreaks confirmed in the past decade alone [6].

Controlling dengue fever relies heavily on the suppression of *Ae. aegypti* populations. A wide array of vector control strategies is available, including habitat modification, insecticide use, biological control, and genetic methods [7]. However, the effectiveness of these strategies hinges on a thorough understanding of mosquito feeding behavior and ecological patterns. Incorporating data on seasonal vector abundance and host-feeding preferences is essential for designing targeted and sustainable interventions [2]. Seasonal abundance patterns, for instance, are linked to arbovirus persistence, transmission, and spread [8], while blood meal profiling reveals key insights into host use and potential reservoir species involved in disease dynamics [9].

Advances in high-throughput DNA sequencing technologies have revolutionized the identification of blood meal sources in hematophagous insects, allowing for the simultaneous detection of multiple host species in mixed samples. These methods have been successfully applied to arbovirus vectors [9,10], but their adoption remains limited in tropical regions where arboviral diseases are most prevalent, primarily due to technical and financial constraints. Cost-effective solutions, such as DNA pooling, have emerged as viable alternatives for large-scale blood meal analysis. Pooled sample sequencing, often coupled with metagenomic or metabarcoding approaches, allows for the efficient detection of multiple vertebrate hosts in a single sequencing run [11].

In Kenya, the continuous transmission of several arboviruses—including dengue [12,13,14], chikungunya [15,16,17], West Nile virus, Rift Valley fever virus [18], and O’nyong-nyong virus [19,20]—has been reported in both Ukunda (Coastal Kenya) and Kisumu (western Kenya). *Ae. aegypti*, the dominant vector in these regions, breeds year-round and exploits a variety of artificial containers such as tires, buckets, drums, and other domestic receptacles for oviposition [2,21,22,23,24,25].

In this study, we investigated temporal fluctuations in *Ae. aegypti* abundance in Ukunda and Kisumu and profiled their vertebrate blood meal sources using amplicon-based metabarcoding from pooled mosquito DNA. Our aim was to deepen understanding of the ecology and feeding dynamics of *Ae. aegypti* in Kenya and explore the implications for arboviral disease transmission and control.

## 2. Materials and Methods

### 2.1. Study Sites

**Figure 1:**
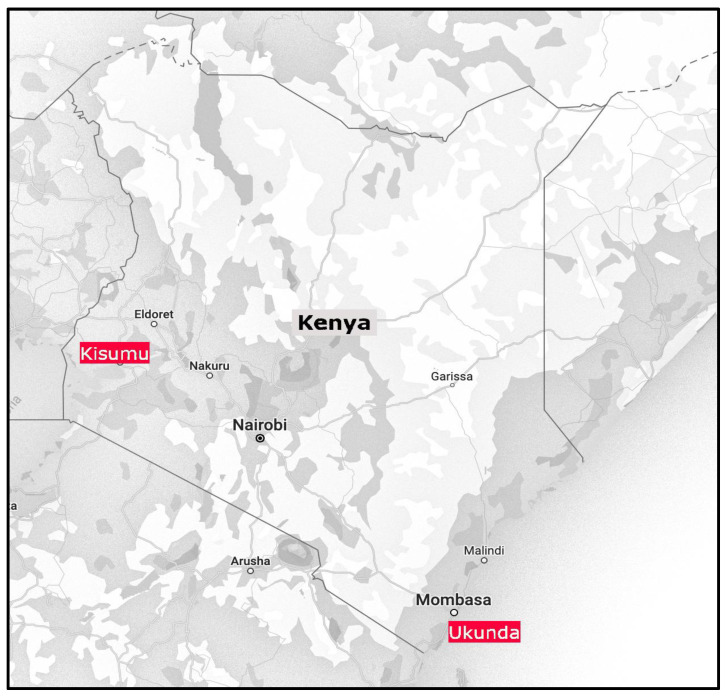
Map showing the sites sampled in coastal and western regions of Kenya. Source: [27]. Ukunda and Kisumu [both highlighted in red] were the study sites in coastal and western Kenya, respectively.

This study tested engorged female mosquitoes that were initially collected as part of a longitudinal study. The study involved the year-round sampling of vector populations from 2020 to 2022. Collections were conducted in the Gombato area of Ukunda town and the Migosi area of Kisumu town, located in the coastal and western regions of Kenya, respectively (Figure 1). Ukunda (4°16′38.8992″ S, 39°34′9.0012″ E) is an urban site, located approximately 30 km south of the port city of Mombasa. It is a rapidly growing urban center with a population of 96,868 [26]. Almost half of the population live in overall poverty with small business enterprises. Fishing and tourism are the main economic activities. Rainfall peaks occur twice in a year; March to June experiences long rains, while short rains are observed from October to December. The average total rainfall is about 1060 mm per year with annual mean temperatures of 28.5 °C ranging from 23 °C to 34 °C, and an average relative humidity of 74%. Kisumu (0°5′15.2247800″ S, 34°46′22.328400″ E) is also an urban site located on the shores of Lake Victoria and the headquarters of Kisumu County. It has an overall population of 397,957 people according to the 2019 population census [26]. Some sections of the city are well developed and maintained, while others are poorly drained and peri-urban, where mosquitoes mostly breed. The rainfall in Kisumu is 966 mm per year with an average humidity of 63%. Rainfall is bimodal with short rains occurring between September and November and the long rains between March and May. It has an average year-round temperature of 23.1 °C ranging from 16 °C to 33 °C [2].

### 2.2. Mosquito Collection Methods

The two study sites were divided into eight 200 × 200 m zones. Mosquitoes were collected in each of the zones using Biogents sentinel traps (Biogents AG, Regensburg, Germany) and Prokopack aspirators (John W. Hock., Gainesville, FL, USA) from October 2020 to January 2022.

Mosquito collection by Biogents sentinel (BG) traps was completed once every two months in each of the eight zones per study site. The collection effort involved setting two BG traps, one residential (house) and one non-residential space in each zone for five consecutive days. During the 16-month period, eight trapping efforts each for a residential and non-residential space were made. For residential trapping, the trap was placed outdoors in a secure location close (no more than 5 m) to the randomly selected residential house in each zone. One non-residential space was selected for BG trapping from a range of available suitable spaces in each zone. The non-residential spaces with secure locations available in the two study sites for BG trapping included gardens, open spaces, uninhabited houses, yard shops, banana stands, a field, near an open market, a church, and a goat shed. The suitability of a trapping site was assessed mainly based on the security of the trap. Baiting, setting, and the recovery of mosquitoes in the residential and non-residential spaces followed the procedures described by Ndenga et al. and Peña-García et al. [24,27]. Briefly, CO_2_ was produced from a mixture of 35 g yeast (Angel Yeast Co., Ltd., Cairo, Egypt) and 500 g of sugar in 5 L of water. This mixture was replaced on the third day after setting up the experiment. On each day of sampling at about midday (every 24 h), in addition to retrieving trapped mosquitoes, the battery was replaced with a charged one.

Mosquito collection by Prokopack aspirator was completed monthly in each of the eight zones per study site [27]. Prokopack aspiration was performed in the outdoor domestic environment of eight participating households and eight non-households’ structures or spaces in each of the study zones. During the 16-month period, 16 trapping efforts each for a residential and non-residential space were made. Prokopack aspiration was performed within 10 meters around the selected house and the non-household space from 0900 to 1500 h [24,27,28]. At each site, two Prokopack aspirators were operated simultaneously in household and non-household spaces for 10 min each.

Mosquitoes trapped by both methods were transported in a cooler box with ice packs. Samples from the Kisumu site were taken to the insectaries at the Kenya Medical Research Institute, Centre for Global Health Research station in Kisian, Kisumu County. Samples from the Ukunda site were taken to the Vector Borne Disease Control Unit at Msambweni County Referral Hospital in Kwale County. The collected mosquitoes were identified by morphology using taxonomic keys [29,30] and then sorted by abdomen status. Blood-fed *Ae. aegypti* mosquitoes were placed in separate vials and stored at **4 °C** awaiting DNA extraction.

### 2.3. DNA Extraction

DNA was extracted from 35 and 25 blood-fed *Ae. aegypti* mosquitoes collected from the Ukunda and Kisumu sites, respectively. To characterize the abdomen contents of blood-fed and half-fed *Ae. aegypti* mosquitoes, individual blood fed mosquito abdomens were separated from the rest of the body using a sterile scalpel. Each separated abdomen was transferred into a sterile 1.5 mL microcentrifuge tube and triturated in 500 μL of phosphate buffered saline (PBS). DNA was then extracted using the Quick-DNA^TM^ Miniprep kit (Zymo Research, Orange, CA, USA) according to the manufacturer’s instructions. The quality and quantity of DNA was confirmed with NanoDrop^®^ spectrophotometer (Thermo Fisher Scientific, Waltham, MA, USA), and 1% agarose gel electrophoresis ran at 150 volts stained with SYBR green and visualized using a UV transilluminator.

Pooling for group test involved DNA quantification by Qubit^TM^ 4 fluorometer (Thermo Fisher Scientific, Waltham, MA, USA) and normalization to ensure equal amounts of DNA from each mosquito abdomen were used [31]. Equal volumes of DNA were combined into two separate pools corresponding to the study sites, Ukunda and Kisumu. The two pools of mosquito DNA and one pool of human DNA were sent to Inqaba Biotec (Pretoria, South Africa), a commercial NGS service provider, for sequencing.

### 2.4. Next Generation Sequencing [NGS]

For the detection of blood source, a fragment of cytochrome b *(cytb)* gene was amplified using universal vertebrate primers Cytbvert1D(R) CCATCCAACATYTCADCATGA and Cytbvert2D(F) GCHCCTCAGAATGATATTTGK [32]. Amplification was performed in 100 μL of a solution containing 67 mM Tris (pH 8.8), 6.7 mM MgSO_4_, 16.6 mM NH4_2_SO_4_, 10 mM 2-mercaptoethanol, each dNTP at 1 mM, each primer at 1 μM, genomic DNA (10–1000 ng), and 2–5 units of Taq polymerase. Each cycle of the polymerase chain reaction consisted of initial denaturation for 2 min at 94 °C, followed by 40 cycles of denaturation for 30 s at 94 °C, hybridization for 30 s at 50 °C, extension for 2 min at 68 °C, and a final extension for 5 min at 68 °C.

The resulting 300 bp amplicons were purified and end-repaired, and illumina-specific adapter sequences were ligated to each amplicon using the NEBNext^®^ Ultra™ II DNA Library Prep Kit for Illumina^®^ and the DNA pools were indexed with the NEBNext^®^ Multiplex Oligos for Illumina^®^ (New England Biolabs, Ipswich, MA, USA) following quantification. Another purification was performed with AMPure XP beads (Beckman Coulter, Miami, FL, USA). Amplicons were then quantified using Qubit^®^ dsDNA HS Assay Kit (Thermo Fisher Scientific, Waltham, MA, USA) and sequenced on illumina’s MiSeq platform using a MiSeq v3 (600 cycle) kit following the manufacturer’s protocol.

### 2.5. Data Analysis

The raw sequence reads were filtered and trimmed to a minimum quality score of Q20 and length of 80 bp, and then merged using fastp v0.19.5 [33]. The pre-processed reads were clustered into operational taxonomic units (OTUs) at 99% sequence identity using CD-HIT [34]. Each OTU was used to query the sequence against the NCBI non-redundant (“nr”) nucleotide database. The resulting hits were filtered to retain those with a similarity match of ≥96% identity and a minimum of 100 bp reads between the reference sequence and the queried sequence. A Kruskal–Wallis statistical analysis test was performed to compare the distribution of *Ae. aegypti* infestation in the two study sites by months and/or seasons. Statistical analysis and display plots were performed on R environment.

## 3. Results

### 3.1. Mosquito Abundance and Spatial–Temporal Distribution

**Figure 2:**
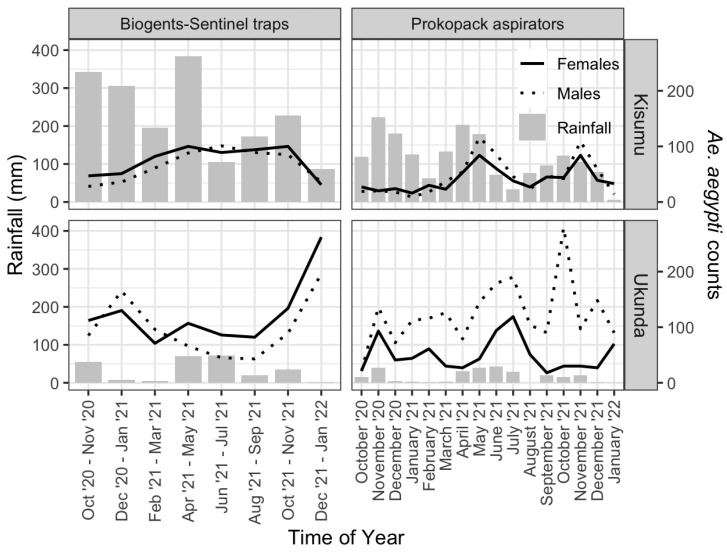
Temporal variation of rainfall (bars) and counts (lines) for female (solid lines), and male (dotted lines) *Ae. aegypti* mosquitoes, caught by Biogents-Sentinel (**left** plots) and Prokopack aspirator (**right** plots) traps in Kisumu (**top** row) and Ukunda (**bottom** row) sites over the study period.

In total, 64,360 mosquitoes were collected using the two mosquito sampling methods during the 16 months. Most of the collected mosquitoes were *Culex* spp. (88.9%; 57,221), followed by *Aedes aegypti* (10.9%, 7035) and ‘others’ (0.2%; 104/64360). The ‘others’ category comprised *Aedes simpsoni*, *Anopheles gambiae* s.l., *An. funestus,* and *Toxorhynchites*. Overall, Prokopack aspirators trapped more *Ae. aegypti* than BG traps. BG traps collected more *Ae. aegypti* in Ukunda sites (1769) than Kisumu sites (1118), but the difference was not significant (Kruskal–Wallis test, χ^2^ = 0.002, df = 1, *p* = 0.966). Prokopack aspirators collected significantly more *Ae. aegypti* mosquitoes in Ukunda sites (2784) compared to Kisumu sites (1364) (Kruskal–Wallis test, χ^2^ = 16.084, df = 1, *p* < 0.0001). BG traps collected more females than males (55% vs. 45%), while Prokopack aspirators were biased towards males (63% vs. 37%). There were no clear temporal trends over time for the number of *Ae. aegypti* collected by BG traps in both sites across eight sampling periods (Kruskal–Wallis test, χ^2^ = 11.382, df = 7, *p* = 0.123) (Figure 2). The abundance of *Ae. aegypti* mosquitoes collected by Prokopack aspirators varied seasonally (Kruskal–Wallis test, χ^2^ = 10.737, df = 3, *p* = 0.0132). The least number of *Ae. aegypti* was recorded during the dry season (January–March) and the highest number was reported during the long rain season (April–June). The temporal distribution pattern of *Ae. aegypti* collected by Prokopack aspirators corresponded with rainfall patterns in both sites (Figure 2). A total of 35 blood-fed *Ae. aegypti* mosquitoes were collected from the Ukunda site, while 25 were collected from the Kisumu site across both traps.

### 3.2. Vertebrate Food Sources

In our study, a diverse range of *Ae. aegypti* blood meal hosts were identified across the two study sites, Ukunda and Kisumu. In total, 15 vertebrate species were identified as blood meal sources by next generation sequencing from the two *Ae. aegypti* mosquito pools (Table 1). Of the 15 vertebrates, 8 were mammals, 5 aves, 1 reptile, and 1 blenny fish (Actinopterygii). Overall, humans were the preferred blood meal source for *Ae. aegypti* at a relative abundance of 86.6%. Other key mammalian blood meal sources were *Cervus elaphus* (red deer) and *Ovis species* (sheep), contributing 12.3% and 0.7%, respectively, at the Ukunda site only. Aves, reptiles, and fish (Actinopterygii) represented 1.1% of the identified blood meal hosts (Table 1). The human DNA positive control yielded 100% identification as *Homo sapiens*, thereby confirming the accuracy, sensitivity, and specificity of the methodological pipeline.

At the Ukunda site, hosts included chimpanzee (*Pan troglodytes*), chicken (*Gallus gallus*), turkey (*Meleagris gallopavo*), pheasant (*Phasianus colchicus*), goat (*Capra hircus*), cow (*Bos taurus*), red deer (*Cervus elaphus*), sheep (*Ovis* species), wild yak (*Bos mutus*), quail (*Cortunix japonica*), crow (*Corvus moneduloides*), lizard (*Acanthodactylus* cf. *cantoris*), and blenny fish (*Parablennius sanguinolentus*). In the Kisumu site, identified hosts included gorilla (*Gorilla gorilla*), chimpanzee (*Pan troglodytes*), chicken (*Gallus gallus*), turkey (*Meleagris gallopavo*), and pheasant (*Phasianus colchicus*).

There were almost twice as many hosts in Ukunda compared to Kisumu (Table 1). Gorilla was only identified in Kisumu, while lizard and blenny fish were only found in Ukunda. Uniquely, apart from aves, no other domestic animals were identified from mosquitoes collected in Kisumu. Most of the vertebrates identified commonly range in the respective environments save for the deer, gorilla, chimpanzee, and wild yak, which are not typically in range in the study area.

## 4. Discussion

Our main findings show that humans (87–96%) are the preferred blood meal vertebrate hosts for *Ae. aegypti* mosquitoes in the two study communities. A wide range of other vertebrate hosts were also identified, demonstrating that the anthropophilic nature of this mosquito persists where other vertebrate choices are available [35,36,37]. The temporal abundance of *Ae. aegypti* mosquitoes was also associated with rainfall patterns. While vertebrate host preferences were comparable between coastal and western regions, significantly more mosquito vectors were collected in coastal Kenya. This observation may partly explain the differences in dengue virus burden in coastal and western regions [6,12,13,14,38,39,40,41,42].

In both towns, the abundance of mosquitos collected by Prokopack aspirators correlated with rainfall patterns with clear peaks during the long rains (May–July) and short rains (October–December). The observed seasonal trend from Prokopack aspirators is consistent with a similar longitudinal study in the study area [24]. Heavy rainfall usually provides a favorable environment for mosquito breeding [2,43,44]. An almost similar trend was observed in dengue fever cases in the study area [38], signifying the key role played by rainfall pattern in the transmission dynamics of dengue virus. However, studies in Ecuador and Australia have found that households with unreliable water services are more likely to harbor mosquitoes, as stored water during droughts provides ideal breeding sites for *Aedes aegypti* [45,46].

The significantly higher densities of *Ae. aegypti* reported in Ukunda compared to Kisumu can be attributed to the higher breeding activity in the coastal region, as described by Ngugi et al. [2], and may explain the highly frequent dengue fever outbreaks on the coast [6]. The correlation between rainfall, water storage practices, and increased mosquito abundance highlights the importance of integrated vector management. Public health efforts should prioritize educating communities on proper water storage, including covering and maintaining containers, to reduce breeding. Sustainable water management systems can further minimize reliance on open storage and curb mosquito proliferation.

Prokopack aspirations in the outdoor spaces were extended to other non-household environments around the houses that are typically vegetated [27]. The collection of males resting in the vegetated environment or while actively seeking females for mating may explain the biased male Prokopack aspiration [47]. No seasonal correlations were noted between the number of *Ae. aegypti* collected by BG trap and rainfall patterns. Unlike Prokopack aspirators, which trap mosquitoes of all physiological states and behavioral stages in a population, BG traps typically target host-seeking and blood-fed female mosquitoes [36,48]. The performance of the BG traps is reported to be affected by whether it is CO_2_-baited and the source of the CO_2_ [49,50]. A recent study reported dry ice may be a better source of CO_2_ for mosquito trapping compared to yeast-derived sources [49]. The poor performance of BG traps in the current study remains undetermined, but the use of yeast-derived sources of CO_2_ is a plausible explanation.

The strong preference for human blood observed in this study has direct implications for arbovirus transmission dynamics. *Ae. aegypti* is the primary vector for arboviruses such as dengue, chikungunya, Zika, and yellow fever, which are transmitted predominantly through human–mosquito–human cycles [37]. In highly urbanized areas like Kisumu, where non-human blood meal hosts are scarce, mosquitoes are more likely to rely almost exclusively on human hosts. This behavior intensifies the potential for virus amplification within human populations, as the frequency of infectious bites increases due to repeated contact between humans and *Ae. aegypti*.

Contrasting this study with findings from other regions in Kenya, Agha et al. [35] reveals the role of host availability in shaping feeding behavior and subsequent arbovirus risks. They reported that, in peri-urban Mombasa and Kisumu, domestic animals (e.g., cattle, goats, dogs, and cats) constituted significant proportions of the blood meal sources (36% and 62%, respectively). In such peri-urban environments, the availability of alternative non-human hosts likely reduces the intensity of mosquito–human interactions, thereby potentially mitigating the risk of arbovirus transmission to humans. However, in urban Kisumu, where human density is high and domestic animal density is low, *Ae. aegypti*’s near-exclusive reliance on human blood meals amplifies the arbovirus transmission risk.

Urbanization in Kisumu significantly exacerbates arbovirus transmission risks through several interconnected factors. Firstly, the high human density in densely populated urban areas provides an abundant and consistent blood source for *Ae. aegypti*, enabling uninterrupted transmission cycles [51]. Secondly, the scarcity of non-human blood meal sources in urban environments forces *Ae. aegypti* to rely predominantly on human hosts. This limited access to alternative hosts increases the likelihood of virus transmission and the occurrence of outbreaks [37,52]. Furthermore, the persistence and continuous circulation of arboviruses such as dengue and chikungunya in both *Ae. aegypti* mosquitoes and human populations highlight the ongoing transmission risks in urban Kisumu. Studies have shown that, in urbanized settings, the absence of animal reservoirs sustains a cycle of infection, propagating arboviruses within human populations and heightening the potential for large-scale outbreaks [21,38,40,53,54,55]. The findings align with the global understanding of *Ae. aegypti* as a highly adaptable urban vector. Urban expansion in Kisumu without adequate vector control interventions, such as eliminating breeding sites or improving waste management, provides ideal conditions for *Ae. aegypti* proliferation and virus transmission [52]. Additionally, the absence of alternative blood meal hosts could compel *Ae. aegypti* to rely solely on humans, increasing the risk of large-scale outbreaks.

Some other blood meal source identification studies in Kenya include Karisa et al. [56], who used a direct enzyme-linked immunosorbent assay (ELISA) to identify the blood meal sources of Afrotropical malaria vectors along the coast, finding that nearly half had fed on cattle, followed by goat, human, and mixed hosts, with few samples unidentified. Omondi et al. [57] applied high resolution melting (HRM) analysis of *cyt b* and 16S rRNA PCR products, followed by sequencing, to identify 33 vertebrate blood meal hosts in the Lake Victoria and Lake Baringo regions. The hosts included humans and 8 domestic, 6 peridomestic, and 18 wildlife species. Also, during the 2006–2007 Rift Valley Fever outbreak, Lutomiah et al. [58] analyzed blood-fed mosquitoes using the PCR amplification of *CO1* and *cytb* genes, identifying goats, cattle, donkeys, sheep, humans, frogs, duikers, camels, and some unidentified hosts.

Blood meal source identification studies from other tropical areas include Katusi et al. [59], who assessed malaria vector blood meals monthly in Tanzania’s Kilombero Valley using ELISA. They found bovine as most common, followed by human, dog, goat, and sheep. Also, in Cape Verde, Gonçalves et al. [60] used direct ELISA to link mosquito species to hosts, noting preferences for humans, dogs, chickens, and mixed feeds, mainly involving two hosts.

From the foregoing, mosquitoes feed on diverse hosts, which include humans, livestock, wildlife, and poultry, significantly increasing the risk of zoonotic disease transmission. The high occurrence of human blood meals in our study indicates elevated human–vector interaction, raising the likelihood of outbreaks. Their flexible feeding behavior enables pathogens to persist even when human contact is limited, complicating control efforts. Feeding on multiple hosts also facilitates cross-species transmission, potentially driving the emergence of new diseases. These insights highlight the importance of integrated vector management and a One Health approach. Effective mitigation demands targeted interventions, combining vector control with public education. Strengthening surveillance and reducing *Ae. aegypti* populations can help curb arbovirus spread, while community-level actions, such as eliminating mosquito breeding sites, are vital. Integrating urban planning with health strategies ensures that human, animal, and environmental health are jointly protected.

Domestic animals such as chicken, turkey, goat, cow, and sheep are common in Ukunda and its environs. Local communities often rear poultry and livestock for subsistence and commercial purposes, providing readily available blood meal sources for mosquitoes. Similarly, in Kisumu, chickens are widely domesticated, with rural and peri-urban households rearing them for food and income [61,62]. Their abundance and outdoor living conditions make them accessible hosts for mosquitoes. Turkeys, though less common than chickens, are reared on a small scale in Kisumu and surrounding areas, reflecting a growing interest in poultry diversity to meet nutritional and economic demands in Kenya [63].

The presence of pheasant and quail in Ukunda, although these species are native to other regions, may be attributed to domestication or farming activities. Quail farming has gained popularity in various parts of Kenya. Similarly, in Kisumu, pheasants may represent domesticated individuals or related native bird species such as guinea fowl (*Numida meleagris*), which are common in the region. The detection of chimpanzee and gorilla is likely due to misidentifications arising from database limitations. These non-native primates, indigenous to Central and West Africa, may phylogenetically overlap with native Kenyan species such as monkeys and baboons, which are abundant in these areas [64,65].

Other non-native species detected in Ukunda included red deer (*Cervus elaphus*), wild yak (*Bos mutus*), crow (*Corvus moneduloides*), lizard (*Acanthodactylus* cf. *cantoris*), and blenny fish (*Parablennius sanguinolentus*). The identification of these species can also be attributed to database insufficiency, with actual hosts likely being related native species with similar sequences. For instance, tropical species are underrepresented in public reference databases, and sequencing errors or PCR chimeras can lead to the classification of spatially infeasible taxa in dietary metabarcoding experiments [66,67,68]. Notably, red deer was detected at a relatively high representation (12.3%), which warrants further scrutiny.

The identification of blenny fish as a blood meal source for *Ae. aegypti* at Ukunda is a novel finding. This result aligns with reports of *Aedes baisasi* feeding on fish in brackish mangrove habitats [69]. This study from the Ryukyu Archipelago revealed that air-breathing fish such as mudskippers and four-eyed sleepers play a significant role in the diet of mangrove-inhabiting mosquitoes. Similarly, our detection suggests that *Ae. aegypti* may opportunistically feed on aquatic species, likely during instances when these fish surface or interact with shoreline, estuary, or dam environments. This finding expands the documented host range for *Ae. aegypti*, highlighting its adaptability to diverse ecological settings. Further research is necessary to clarify the extent of fish as a blood meal source and its implications for mosquito population dynamics and vectorial capacity in coastal areas.

The identification of geographically disparate and non-indigenous species such as chimpanzees, gorillas, red deer, and wild yak, albeit in low representation (<0.2% relative abundance), underscores the limitations of high-throughput sequencing in host identification. False positives can arise from PCR and sequencing errors, leading to chimeric sequences that are often classified as spatially infeasible taxa [66]. Database insufficiencies, particularly the underrepresentation of tropical species, further complicate accurate identification [67,68]. These challenges highlight the need for improved reference databases and rigorous validation methods in dietary metabarcoding studies.

The limitations of metagenomics present several challenges that can impact its accuracy and reliability. A significant drawback is the potential for misclassification, where errors in taxonomic assignment may occur due to incomplete or biased reference databases, resulting in the misidentification of blood meal sources [70,71]. Furthermore, unlike PCR or quantitative PCR, metagenomics generally produces qualitative or semi-quantitative data, which limits its ability to precisely estimate the relative proportions of different blood sources [72]. Another key challenge arises from the digestion of blood meals within the mosquito’s midgut, which often leads to DNA degradation. Under such circumstances, metagenomics may have difficulty accurately identifying degraded DNA fragments, whereas PCR, which specifically targets shorter and well-defined DNA sequences, proves more effective for such analyses [10,73,74].

Nonetheless, the metabarcoding data provide a useful overall snapshot of vertebrate host repertoire as well as host preference via relative abundance, even if quantitative comparisons between samples, sites, and seasons were limited by sample pooling. Based on the demonstrated utility of an affordable metagenomic approach to profile blood meal sources, it is recommended that future research and surveillance programs in vector biology and disease control adopt this cost-effective metagenomic technique. By implementing this approach, researchers can efficiently and accurately identify blood meal sources across a wide range of ecological contexts, improving our understanding of disease transmission patterns.

The findings of this study significantly contribute to advancing the understanding of seasonal variation in abundance of *Ae*. *aegypti* and blood meal hosts in coastal and western Kenya by highlighting key ecological, seasonal, and behavioral patterns of *Ae. aegypti*, a primary vector for arboviral diseases such as dengue, chikungunya, and Zika. The observed variation in *Ae. aegypti* abundance, which corresponds to seasonal rainfall patterns, offers critical insights into the vector ecology. This study recorded the highest mosquito abundance during the long rainy season, with numbers declining in the dry season. These findings align with other studies by Ngugi et al. and Mweya et al. [2,75], which report that *Ae. aegypti* populations increase with rainfall due to the creation of breeding habitats such as stagnant water in containers and puddles. Understanding these seasonal trends can aid in developing predictive models for mosquito population surges, thereby enhancing the timing of vector control interventions. The seasonal abundance data can also be used to guide public health agencies in preemptive measures, such as intensified mosquito control during peak seasons. This is particularly important in coastal regions like Ukunda, where *Ae. aegypti* mosquitoes were more abundant (64.7% of total collected).

The identification of a wide range of blood meal hosts across both sites provides valuable insights into the feeding behavior and adaptability of *Ae. aegypti*. The ability of *Ae. aegypti* to feed on multiple hosts, including humans, livestock, birds, and even reptiles, demonstrates its opportunistic nature. This finding underscores the role of *Ae. aegypti* as a bridge vector that can transmit pathogens between animals and humans, increasing the risk of zoonotic arboviral outbreaks. At the Ukunda site, the identification of both domestic and wild animal hosts, such as chicken, cow, and primates, highlights the complexity of the transmission cycle and the potential reservoir of hosts. This corroborates findings by Mwalugelo et al. [76] that domestic animals can act as alternative blood sources for mosquitoes when human hosts are limited. In Kisumu, the narrower range of blood meal hosts may reflect differences in land use and human density, with urban settings providing limited access to wild hosts compared to peri-urban or rural sites.

This host diversity also suggests that mosquito control measures focusing solely on humans may be insufficient and highlights the need for integrated approaches that consider animal reservoirs, particularly in areas like Ukunda with substantial wildlife interactions. Effective public health mitigation strategies are crucial to control *Ae. aegypti* populations and reduce arboviral transmission risks in Ukunda (coastal) and Kisumu (western Kenya). The findings on the seasonal variation and diverse blood meal hosts of *Aedes aegypti* provide essential insights into the vector’s ecology and transmission dynamics. The seasonal abundance highlights the need for targeted vector control interventions, especially during the rainy season. Furthermore, the opportunistic feeding behavior observed in Ukunda and Kisumu necessitates integrated approaches that consider both human and animal hosts in arbovirus control. By implementing robust public health strategies, such as source reduction, enhanced surveillance, and community engagement, the risk of arboviral infections can be effectively mitigated in these regions.

## 5. Conclusions

Our results provide key insights into *Aedes aegypti* abundance, seasonality, and host preference in coastal and western Kenya. Over 16 months, 64,360 mosquitoes were collected, with *Culex* spp. comprising 88.9% and *Ae. aegypti* 10.9%. Prokopack aspirators, particularly effective in Ukunda, captured more *Ae. aegypti* and reflected seasonal trends, peaking during the long rains. The blood meal analysis of 60 fed *Ae. aegypti* showed a strong human preference (86.6%), indicating high disease transmission potential. Fifteen vertebrate species were identified, including eight mammals, five birds, one reptile, and one fish (blenny fish). Host diversity was greater in Ukunda, though some unexpected species likely reflect database limitations. The findings highlight Prokopack effectiveness, rainfall’s role in mosquito dynamics, and significant human–mosquito contact. These findings can be exploited in the development of vector monitoring and control tools.

## Figures and Tables

**Table 1:** Relative abundance (RA) and identity levels (ID) of vertebrate blood meal sources for *Ae. aegypti* in Ukunda and Kisumu towns.

Organism	Common Name	Species	Kisumu		Ukunda	
			RA (%)	ID (%)	RA (%)	ID (%)
Mammals	Human	*Homo sapiens*	95.88	100.00	86.55	100.00
	Goat	*Capra hircus*	0.00	-	0.06	99.72
	Cow	*Bos taurus*	0.00	-	0.09	99.44
	Red deer	*Cervus elaphus*	0.00	-	12.28	95.24
	Gorilla	*Gorilla gorilla*	0.05	91.64	0.00	-
	Chimpanzee	*Pan troglodytes*	0.11	97.21	0.01	97.21
	Sheep	*Ovis species*	0.00	-	0.72	100.00
	Wild yak	*Bos mutus*	0.00	-	0.03	100.00
Aves	Chicken	*Gallus gallus*	0.23	100.00	0.13	100.00
	Turkey	*Meleagris gallopavo*	3.65	97.17	0.02	97.17
	Quail	*Cortunix japonica*	0.00	-	0.04	97.00
	Crow	*Corvus moneduloides*	0.00	-	0.02	93.00
	Pheasant	*Phasianus colchicus*	0.08	100.00	0.02	100.00
Reptiles	Lizard	*Acanthodactylus* Cf. *cantoris*	0.00	-	0.02	98.02
Fish	Blenny fish	*Parablennius sanguinolentus*	0.00	-	0.01	98.61

-: Not applicable.

## Data Availability

Data supporting the conclusions of this article are provided within the article.

## Abbreviations

The following abbreviations are used in this manuscript:

CD-HIT	Cluster Database at High Identity with tolerance
*CO1*	Cytochrome c oxidase subunit 1 gene
*Cytb*	Cytochrome b
DNA	Deoxy-ribonucleic acid
ELISA	Enzyme-linked immunosorbent assay
HRM	High resolution melting
MiSeq	Next generation sequencing platform for genomic analysis
NACOSTI	National commission of science, technology and innovation
NCBI	National Centre for Biotechnology Information
NGS	Next generation sequencing
OTU	Operational taxonomic unit
PCR	Polymerase chain reaction
